# Gonorrheal Conjunctivitis

**Published:** 1912-08

**Authors:** 


### Gonorrheal Conjunctivitis.

From the University Eye-Clinic at Jena, Prof. D. W. Stock (Director), come very excellent reports from Dr. G. A. Hegner, Senior Clinical Assistant, upon the results obtained from the use of Syrgol in conjunctival inflammation, especially gonorrheal conjunctivitis.

The favorable reports of Kollbrunner regarding the use of Syrgol in specific urethritis induced the ophthalmologists at Jena to make experiments with the new preparation. Hegner states that their results have been so gratifying that Syrgol is looked upon by them as a most valuable addition to the various means of treating suppurative diseases of the conjunctiva. He says that, where there is thickening of the eyelid with extreme oedematous swelling and the tissues became so hard as to render it difficult to inspect the diseased structures in order to confirm the diagnosis, treatment should be given with the purpose of allaying the inflammation and producing the swelling of the lid. "Protargol, Sophol and Argyrol have in the past proved beneficial, but since our experience with this new salt, Syrgol, we regard it is superior in its ultimate results."—Hegner.

Syrgol is a brownish-black, odorless colloidal oxide of silver. Physically it consists of shining crystalline scales which dissolve in two parts of water. A 5 per cent solution is almost painless and does no damage to the cornea.

In twenty cases of gonorrheal conjunctivitis, he reports that gonococci disappeared from the secretions in a short time and speedy recovery took place in every instance. Three exceptionally severe cases are reported in detail, speedy recovery resulting in

### Summer Ailments

**involving the gastro-intestinal tract or the circulatory system, are especially amenable to**

### Gray's Glycerine Tonic Comp.

**This well-known remedy has the great advantage of never being contraindicated during the heated season, as are cod liver oil and many other tonics. Therefore, it may be given throughout the year without a question as to its therapeutic fitness.**

PURDUE FREDERICK CO.
298 BROADWAY, NEW YORK

each instance. In the three cases described the most noteworthy feature was the rapid disappearance of the gonococci and the prompt subsidence of inflammation.

Good results were also observed in many cases of ophthalmia neonatorum. By using Syrgol healing took place usually in about a week. In two cases recovery took place in four days, and seldom was it necessary to continue treatment longer than two weeks. An interesting fact that he mentions was that two cases which were not doing well previously showed rapid improvement when transferred to the clinic where Syrgol was employed.

Syrgol proved of much service also in cases of conjunctivitis following operation for cataract. Favorable results were obtained also in cases of inflammation of the lachrymal ducts. He mentions a patient suffering from an acute dacryocystitis in which there was swelling and considerable redness, together with feeling of pressure over the duct. The sac was washed out thoroughly with a 1 per cent solution of Syrgol and complete recovery followed in eight days. A similar result was obtained in a case where there was a purulent discharge from the lachrymal sac, but no inflammation present. Two such cases, of course, are not sufficient to enable one to draw positive conclusions, but they certainly indicate that good results in both acute and chronic inflammations of the lachrymal sac may be obtained by irrigation with Syrgol.

The manner of applying the remedy is quite simple. In acute cases of blenorrhea a 5 per cent solution is dropped into the conjunctival sac from two to three times a day, and the eye is bathed frequently with a solution of boracic acid in order to wash away accumulated secretions. In some cases it may be found advisable to use a 2 per cent solution.

The treatment of gonorrheal conjunctivitis is made easy because of the absence of irritation following the use of Syrgol. Instillation of this remedy in the eye and using an antiseptic solution as a wash is quite often sufficient to effect a cure.

